# What’s in a Surname? Physique, Aptitude, and Sports Type Comparisons between Tailors and Smiths

**DOI:** 10.1371/journal.pone.0131795

**Published:** 2015-07-10

**Authors:** Martin Voracek, Stephan Rieder, Stefan Stieger, Viren Swami

**Affiliations:** 1 Department of Basic Psychological Research and Research Methods, School of Psychology, University of Vienna, Vienna, Austria; 2 Department of Psychology, University of Konstanz, Konstanz, Germany; 3 Department of Psychology, University of Westminster, London, UK; 4 Department of Psychology, HELP University College, Kuala Lumpur, Malaysia; Durham University, UNITED KINGDOM

## Abstract

Combined heredity of surnames and physique, coupled with past marriage patterns and trade-specific physical aptitude and selection factors, may have led to differential assortment of bodily characteristics among present-day men with specific trade-reflecting surnames (Tailor vs. Smith). Two studies reported here were partially consistent with this genetic-social hypothesis, first proposed by Bäumler (1980). Study 1 (*N* = 224) indicated significantly higher self-rated physical aptitude for prototypically strength-related activities (professions, sports, hobbies) in a random sample of Smiths. The counterpart effect (higher aptitude for dexterity-related activities among Tailors) was directionally correct, but not significant, and Tailor-Smith differences in basic physique variables were nil. Study 2 examined two large total-population-of-interest datasets (Austria/Germany combined, and UK: *N* = 7001 and 20532) of men’s national high-score lists for track-and-field events requiring different physiques. In both datasets, proportions of Smiths significantly increased from light-stature over medium-stature to heavy-stature sports categories. The predicted counterpart effect (decreasing prevalences of Tailors along these categories) was not supported. Related prior findings, the viability of possible alternative interpretations of the evidence (differential positive selection for trades and occupations, differential endogamy and assortative mating patterns, implicit egotism effects), and directions for further inquiry are discussed in conclusion.

## Introduction

Readers of fiction will be well aware that some novelists and playwrights expend considerable artistic efforts on the naming of their characters. In literature, character names are often symbolic, meaningful, and semantically laden, rather than random, neutral, or irrelevant [1]. Oftentimes, names––aptly or subtly––reflect salient facets of character personality, physique, or physiognomy, allude to characters’ interrelations, or provide clues to ensuing plot development or a character’s destiny, thereby creating a rich semantic micro-layer within the literary work [2]. Within literary studies, under the label of literary onomastics (i.e., the study of names in fiction) such naming techniques have been recognized and investigated for a long time. Clearly, fictional worlds are under the complete control of their creators, so any name-character correspondences should come as no surprise. But what about such correspondences in the real world? Some work has addressed this question.

Bäumler (1980) [3] advanced a genetic-social hypothesis of differential assortment of bodily frames (somatotypes) among certain surnames in the population. This idea builds on a nexus of three facts (biological, societal, aptitudinal), as follows. First, crucial indicators of physique are substantially to very strongly heritable (height: [4]; body mass index: [5]; muscle strength: [6]). Second, patrilineal surnames (father-child transmission) are transmitted like Y-chromosome markers [7], with low error rates (foremost, adoption or extra-pair paternity; see [8], and in Europe go back at least for 16–20 generations [9]. Quite a number of now widespread surnames originally arose from trades, directly denominating these (e.g., Tailors once *were* tailors, just as Smiths once *were* blacksmiths). For example, initially observed social status differences in English surnames, as evident from medieval sources, appear to have been persisted over centuries [10]. Also, since the Middle Ages, trades were organized as guilds that practiced endogamous (in-group) marriage and apprenticeship [11]. Assortative mating on physical characteristics is common [12] and, when additionally coupled with endogamy, will all the more increase social homogamy (similarity by propinquity). All in all, these interrelated phenomena will lead to above-chance preservation of any heritable traits within lineages. Third, there are obvious physical prerequisites for professions like tailors or blacksmiths, positively selecting for these.

In short, combined heredity of surnames and physique, in tandem with past marriage patterns and trade-specific aptitude and selection factors, may have led to discernible physical differences between present-day men called Tailor vs. Smith. In support of this genetic-social hypothesis, evidence has been presented that Smiths indeed are heavier and bulkier than Tailors, deem themselves more suited for strength-related professions, hobbies, and sports [3], and are overrepresented in strength sports, demanding heavy stature, whereas Tailors are overrepresented in endurance sports, demanding light stature [13, 14].

Although these findings are intriguing in themselves and may give rise to further inquiry along similar lines, they have not yet been independently replicated by others [15]. As replication is the hallmark of scientific progress, we revisited and tested the hypothesis of Bäumler [3] in two studies, employing novel, contemporary data and amended methodologies.

## Study 1

This was a systematic (i.e., modified, amended) replication of Bäumler’s study [3], concerning differences in physique and related aptitude between men with surnames Tailor and Smith. Differences in study characteristics mainly concerned instrumentation (detailed below), but also temporal (1970s vs. 2012), study locale (former West Germany vs. Austria), and survey aspects (mailed vs. telephone interview). Independent replication is crucial for the advancement of empirical knowledge [15, 16]; it is therefore emphasized that the current group of researchers had no connections (such as given through research cooperations and co-authorships) with Bäumler and his co-authors.

### Methods

#### Participants

Participants were a sequential random sample from the population of adult men from Vienna with the German-language equivalents of surnames Tailor and Smith (*N* = 111 and 113), as listed in the Austrian online telephone directory. Groups were comparably aged (see Table 1), with the wide age range (19–75 years) corresponding to that (20–63 years) reported in Bäumler’s study [3]. The predecessor study had group sizes of 110 Tailors and Smiths each; hence, our replication was commensurate. From tabulated results in [3], we calculated the typical effect (mean group difference) as *d* = 0.39 (average across three physique and three aptitude variables; see Table 1). A priori power analysis showed that an independent-groups *t* tests (α = .05, two-tailed) has 82% power to detect an effect of this size with group sizes of 111 and 113; hence, the replication study had sufficient power. The sample was a true random sample drawn from a well-defined underlying population of interest; hence, validity threats arising due to forms of selection bias, as conceivable with non-random sampling frames, seem comparatively low.

**Table 1:** Physique and aptitude differences of Tailors vs. Smiths in Study 1 and in Bäumler (1980) [3] reanalyzed.

Study variables	Tailors		Smiths		*t*	*d*		
	*M*	*SD*	*M*	*SD*		Expected sign	Study 1	Bäumler (1980) [3]^a^
Age	45.3	14.0	44.5	15.9	0.41	None	+0.05	0.00^b^
*Physique variables*								
Height (cm)	179.7	7.1	179.7	7.4	0.03	-	0.00	-0.10
Weight (kg)	84.0	14.9	83.3	12.8	0.40	-	+0.05	-0.29‡
Body-mass index (kg/m²)	26.0	4.6	25.7	3.2	0.56	-	+0.08	-0.30* ^c^
*Aptitude variables* ^d^								
Dexterity (composite)	2.48	0.73	2.55	0.66	-0.77	-	-0.10	NA
Strength (composite)	2.97	0.78	2.77	0.76	2.01	+	+0.26*	NA
Dexterity professions	2.37	1.04	2.49	1.01	-0.82	-	-0.12	-0.26‡
Dexterity sports	2.13	0.84	2.10	0.72	0.28	-	+0.04	NA
Dexterity hobbies	2.95	1.05	3.08	0.99	-0.95	-	-0.13	NA
Strength professions	3.28	1.17	2.82	1.11	3.04	+	+0.40**	+0.62***
Strength sports	3.09	0.96	2.86	0.87	1.86	+	+0.25‡	+0.79***
Strength hobbies	2.55	0.88	2.62	1.01	-0.55	+	-0.07	NA^e^

^a^ Study reanalyzed here via effect size *d*.

^b^ Age-equated groups.

^c^ For solidity (kg/cm; i.e., Quetelet’s index).

^d^ School-grade ratings (1: *Very good*; to 5: *Insufficient*); lower numbers reflect higher self-rated aptitude.

^e^ Effect size not reconstructable. NA = not available (see text for details).

‡ *p* < .10

* *p* < .05

** *p* < .01

*** *p* < .001 (two-tailed; one-tailed tests were throughout calculated in Bäumler’s study [3]).

#### Measures

We developed a total of 12 telephone interview items, closely following materials and intentions in Bäumler’s study [3], but with efforts to harmonize divergent item-response formats and data-analytic procedures evident from the prior study. In detailing Study 1 measures, we briefly refer to Bäumler’s study [3] and, in turn, highlight these differences.

Three domains or areas of life (professions, hobbies, sports; in this order) were queried with four items each. Within each domain, dexterity and fine-motor skills vs. strength and gross-motor skills (i.e., ‘Tailor-typical’ vs. ‘Smith-typical’ traits) were queried with two items each (in alternate order). Individual item responses were averaged to arrive at mean item scores across domains.

In Bäumler’s study [3], participants reported their aptitude for professions, hobbies, and sports in general (*per se*), thus potentially confounding interests, preferences, or mere liking with physical aptitude in particular. To overcome this lack of specificity, we requested participants to explicitly focus on their physical aptitude prerequisites for these activities. Furthermore, Bäumler [3] applied, apparently arbitrarily, different response formats (ratings, rankings, and forced choice) across domains. We harmonized this by having respondents use a uniform, common-sense response format very familiar to them, namely the Austrian school-grade system (1: *Very good*, 2: *Good*, 3: *Satisfactory*, 4: *Sufficient*, 5: *Insufficient*).

The four profession items of Study 1 were aptitude for watchmaker and precision engineer (‘Tailor-like’) vs. butcher and brick-layer (‘Smith-like’), all taken from Bäumler [3], who listed these items as examples, but did not state this domain’s actual item total.

Next, the four sport items concerned archery and billiards vs. weightlifting and rowing, all selected from Bäumler [3], wherein four such items were administered. However, Bäumler [3] had respondents rank-order eight sports, opting then to include only ranks 1 to 4 in the analysis, wherein strength vs. dexterity sports scored 2 vs. 1 point, respectively. That is, these items were collapsed to form a single, bipolar (strength-dexterity) dimension. Consequently, differentiated scores were unavailable, only more or less endorsed aptitude for strength sports.

Finally, the four hobby items were model-making and painting vs. gardening and house alteration, of which in Bäumler’s study [3] the first and third ones were administered, whilst not stating the actual item total. Once more, inconsistent to the above procedures in Bäumler [3], the predecessor study of Bäumler [3] here applied a forced-choice format (between strength-dexterity item pairs), scored the frequency of preferences for one type over the other, but merely reported percentages of preponderance of strength hobbies among Tailors vs. Smiths. Owing to these peculiarities (data aggregation), we refrained from converting this comparison into an effect size (see Table 1).

#### Procedure

All male entries for Vienna of the German-language equivalents of the English surnames Tailor and Smith (i.e., Schneider and Schmied/Schmidt) and the most common spelling variants thereof (*N* = 1194 and 1680, respectively) were extracted from the Austrian online telephone directory (www.herold.at/telefonbuch). The English forms of these surnames (i.e., Tailor and Smith), appearing only sparsely in the telephone directory, were not considered. Within surname groups, the sequence of calls made was determined through random numbers, as assigned to the entry lists through a random-number generator (www.random.org).

Calls (with calling identity provided) were made by a male researcher, trained in telephone interview methods and with call-centre work experience, over the week and usually in the evening. This continued until group sizes of the Bäumler study [3] (110 Tailors and Smiths each) had been reached (see power analysis above). Of 690 telephone subscribers called, 143 (21%) could not be reached. Of the 547 men reached, 214 agreed to take part in the study, yielding a participation rate of 41%. Usual non-eligibility criteria for telephone interviews (underage, hearing impairment, developmental disability) were applied as appropriate.

The caller described the study as scholarly (i.e., not market research), concerned with physical aptitude, making no reference to called parties’ surnames. Telephone interviews proceeded in a standardized manner and on an verbal informed-consent basis, taking about 8 min and comprising 15 questions (the above 12 items, plus age, height, and weight, from which body-mass index, BMI, was calculated as kg/m²). Upon completion, participants were thanked and given an e-mail address for obtaining information on study results.

It may be expected that some of the namebearers Tailor or Smith in the population would be related, which, in turn, would reduce within-group variability through clustering effects in the drawn sample. In the absence of information on the degree of relatedness of the individuals in our sample or in the larger population it was infeasible to make allowance of this. However, since the sampled groups did not constitute a substantial part of the total eligible population (for surname Tailor: 111 male individuals from a population of 1194 male telephone directory entries, or 9.3%; for surname Smith: 113 participants from a population of 1680, or 6.7%), any such effects would be negligible. At any rate, reduced within-group variability would operate against the null hypothesis of no Tailor-Smith group differences, instead favoring the alternative hypothesis of actual group differences. However, support for group differences in Study 1 was modest and partial at best (see below, Results). We therefore conclude that any such clustering effects must indeed have been negligible.

Study 1 was conducted in accordance with the principles laid down in the Declaration of Helsinki (6th revision, 2008) and with institutional guidelines of the School of Psychology, University of Vienna. According to national laws (Austrian Universities Act 2002), effective at the time when Study 1 was carried out, solely medical universities were required to operate research ethics committees, for evaluating and approving basic, clinical, and applied medical research proposal (all of this not applicable for Study 1; therefore, Study 1 was exempt from formal ethical approval). Corresponding to the study design (telephone interviews), informed consent was oral and was given explicitly. Participants could withdraw at any time without consequences (in particular, non-participants were never called again), and participants’ names and phone numbers were never archived or linked with study data. S1 Text contains the standardized text of the telephone intervew, along with the verbal informed consent form, and the S1 Dataset contains the Study 1 data.

### Results and discussion

Dexterity and strength composite measures (6 items each) were internally consistent (sample Cronbach α = .65 and .71; mean item-total correlations: .40 and .45; mean inter-item correlations: .25 and .29; all 30 individual inter-item correlations were positive). Dexterity and strength scores were not related in the whole sample (*r* = -.01), as well as within the Tailors and Smiths subgroups. In Bäumler’s study [3], psychometric properties of the measures were not reported.

The results of tests for Tailor-Smith group differences on the physique and aptitude variables, in juxtaposition with a reanalysis of Bäumler [3], are shown in Table 1. In our data, no Tailor-Smith differences in physique (height, weight, and BMI) emerged (*d* = 0.00, 0.05, and 0.08, respectively). In contrast, in the study data of Bäumler [3], Tailors were shorter, lighter, and less bulky than Smiths (*d* = -0.10, -0.29, and -0.30, respectively). However, the effects reported in Bäumler’s study [3] were small and nominally significant (two-tailed *p* < .05) only for bulkiness. Reanalysis of the results of Bäumler [3] showed that the reported weight difference is significant only with one-tailed testing (employed throughout in Bäumler’s study [3]).

Concerning Tailor-Smith differences in the physique-related aptitude variables included in Study 1, Tailors indeed reported higher aptitude on the dexterity-activities composite, while Smiths showed higher aptitude on the strength-activities composite. Although directionally consistent with the hypothesis in Bäumler [3], both effects were small (*d* = -0.10 and 0.26), and only the latter group difference was statistically significant. Further analyses revealed that higher self-rated aptitude for strength activities among Smiths was evident for professions and sports (*d* = 0.40 and 0.25), but not for hobbies (*d* = -0.07).

Of note, all six effects for Tailor-Smith differences from Study 1 analogous to and thus directly comparable with Bäumler [3] were smaller than those he obtained (see Table 1). Bearing in mind the varied procedural decisions in the earlier study [3] (differing item numbers, response formats, scoring rules, and data-analytic considerations across aptitude domains), such factors may well have contributed to this pattern.

What is more, an additional remark in Bäumler’s study [3] (p. 309) may suggest that only a portion of all survey responses collected was actually analyzed: “The two comparison groups were *equated* for age, and *amounted* to 110 for each group” [italics ours]. In Bäumler [3], postal surveys were sent to all male Tailors and Smiths from the telephone directories of Munich and Nuremberg (1.30 and 0.48 mill. inhabitants, as of 1980), and a very high return rate of about 70% was reported. For comparison, we counted 2874 male Tailors and Smiths in the Vienna telephone directory (1.73 mill. inhabitants, as of 2011). According to population-based surname studies [9], both target surnames rank among the most frequent ones found in Germany and Austria, occurring there with similar prevalences. On this account, the total sample (*N* = 220) in Bäumler [3] seems too small to fit with the above city population figures, especially in consideration with the reported 70% return rate. Selecting responses from a larger data collection to form two equally sized, exactly age-matched groups might introduce any biases, perhaps accidentally favoring the hypothesis tested. We postpone further elaboration of these and other peculiarities of Bäumler’s study [3] and further related prior studies to the general discussion.

## Study 2

This was a direct replication of Bäumler’s studies [13] and [14], wherein data as of 1977 and 1983 from former West Germany were analyzed. We sought to replicate these studies’ evidence for prevalence differences of male namebearers Tailor and Smith among successful athletes of track-and-field events demanding different physiques with new, contemporary, large datasets. In addition, this generalizability test was extended to include a different-language country (the UK).

### Methods

#### Data extraction

We screened men’s 2011 rankings for track-and-field events, as available online, for all German state athletics associations (see S2 Text for the URLs), for the Austrian federal athletics association (www.oelv.at/lists), and for UK athletics rankings (www.thepowerof10.info). Occurrences of surnames Tailor and Smith (in the respective language of the country) in these high-score lists were determined. Spelling variants and appearance of these surnames in double surnames or as constituents of composite surnames (e.g., Blacksmith) were included as well. The lists totalled 7001 entries for Austria and Germany (as same-language countries merged for analysis), whilst 20532 (more than in any prior study; see Table 2) for the UK. These large data collections comprised the respective total population of interest for the research question; hence, validity threats arising due to forms of selection bias, as conceivable with sampling frames that are not population-based, could be ruled out.

**Table 2:** Tailor vs. Smith surname prevalences across sport categories in Study 2 and in reanalyzed prior investigations.

Study	Surname	Sports category (stature)			Study *N*	Cochran-Armitage trend test	
	*N*_cat_	Light	Medium	Heavy		*p*	[95% *CI*]
Bäumler (1984) [13]^a^	Tailor	1.14%	0.22%	0.09%		.000007	-1.42 [-2.22 to -0.72]
	Smith	0.98%	1.23%	1.70%		.066	0.29 [-0.03 to 0.63]
	*N*_cat_	1228	2754	2117	6099		
Stemmler & Bäumler (2003) [14]^a^	Tailor^b^	1.17%	0.69%^d^			.008	-0.53 [-0.94 to -0.13]
	Smith^c^	1.47%	2.01%^d^			.029	0.32 [0.03 to 0.62]
	*N*_cat_	5312	6511^d^		11823		
Study 2 (Austrian/ German sample)	Tailor	0.39%	0.91%	0.64%		.164.	0.28 [-0.13 to 0.68]
	Smith	0.85%	1.16%	1.56%		041	0.31 [0.01 to 0.61]
	*N*_cat_	2836	2753	1412	7001		
Study 2 (UK sample)	Tailor	0.47%	0.43%	0.72%		.306	0.15 [-0.14 to 0.43]
	Smith	1.12%	1.40%	1.61%		.027	0.19 [0.02 to 0.36]
	*N*_cat_	10954	7211	2367	20532		

^a^ Study reanalyzed here.

^b, c^Includes similar surnames (^b^ Shoemaker, Webber; ^c^ Baker, Miller).

^d^ Medium-stature and heavy-stature categories were merged.

*N*_cat_ = *N* per sports category. *p* = exact (permutation test) *p* values (two-tailed), = estimated trend parameter (effect size), with exact 95% *CI*; all from Cochran-Armitage trend tests [19, 20].

Study 2 was conducted in accordance with the principles laid down in the Declaration of Helsinki (6th revision, 2008) and with institutional guidelines of the School of Psychology, University of Vienna. Study 2 comprised analyses of publicly accessible sports statistics and, as such, according to national laws (Austrian Universities Act 2002), was exempt from formal ethical approval. The Study 2 data are displayed in Table 2.

#### Data analysis

From [13], we adopted a three-way categorization of sports, as follows. Light-stature sports (track events only) comprised middle-distance and long-distance races (0.8, 1, 1.5, 3, 5, 10 km races; 10 km road race; 3 km steeplechase; half marathon and marathon). Medium-stature sports (partly track, partly field events) comprised sprints (60, 100, 200, 400 m races), hurdles (60, 110, 400 m), and jump events (high, long, and triple jump; pole vault). Heavy-stature sports (field events only) comprised throw events (discus, hammer, javelin, and shot put) and one combined event (decathlon). As non-individual competitions, relay events were not considered. For the veridicality of such differences in typical somatotype between sport types, as implied by the above categorization, see [17].

According to the hypothesis of Bäumler [3], the frequency of namebearers Tailor (vs. Smith) should decrease (vs. increase) across the above ordered categories (from light-stature to medium-stature to heavy-stature sports). However, the analyses of previous studies did not fully exploit and test the trend information inherent in this data structure. Initially, in [13], the ordinary (Pearson’s) χ² independence test was used to analyze the 2 × 3 contingency table of the two target surnames’ frequencies across the three sports categories. Ignoring the large remainder of non-target surnames collected (>98% of the sample) greatly inflated the effect. At the same time, the trend hypothesis (ordered sports categories) was not accounted for by this test (which is appropriate for unordered categories).

Later, in [18], configural frequency analysis (an exploratory, pattern-detecting method for categorical data) was utilized to reanalyze the data of [13]. This reanalysis used all data, arranged in a doubly-ordered 3 × 3 contingency table (three surname categories: Tailor, all other, Smith; three stature-based sports categories: light, medium, heavy). However, defining Tailors and Smiths as extreme categories and all other surnames in between seems problematic because, with regards to the hypothesis tested, more ‘extreme’ surnames than these may well occur (e.g., surnames like ‘Featherlight’ or ‘Tonweight’ are at least conceivable), but these would incongruously be classified into the middle category of all other surnames.

Finally, in [14], medium-stature and heavy-stature categories were collapsed to contrast this merged category with the light-stature category, again utilizing a χ² independence test. In addition, the data collection of [14] included trade surnames assumed to be similar with regards to the hypothesis (for surname Tailor: Shoemaker, Webber; for surname Smith: Baker, Miller). Hence, these prior studies [13, 14, 18] differed among themselves in important procedural details (surname inclusion and data-analytic approach).

We used the Cochran-Armitage trend test [19, 20], now frequently used in dose-response studies, for a uniform, consistent reanalysis of previously published available data [13, 14], along with our Study 2 data. This directional test is appropriate for singly-ordered contingency tables of size *r*(2)×*c*, wherein the two rows (*r*) constitute binary responses (events vs. non-events) and the columns (*c*) are groups ordered in some way (e.g., dose levels or increasing risk). The Cochran-Armitage trend test has high power to detect a linear trend in the proportion of events to increase or decrease across the ordered groups. In our application of the test, the target surname proportions constitute the events recorded in the binary response variable (Tailor surname vs. all other surnames; or Smith surname vs. all other surnames) and the sports categories constitute the ordered groups (ranging from light-stature over medium-stature to heavy-stature sports). Analyses were conducted in StatXact v4.0.1 (Cytel Inc.) [21]. We report the exact (permutation test method), rather than asymptotic, *p* values (two-tailed) and confidence intervals for the trend parameter (β, estimated via the conditional maximum likelihood method) because of the low target surname rates and unequal frequencies across sport-type cells in the contingency tables analyzed (Table 2). Even with large samples, such disparities may lead to improper asymptotically derived *p* values [22].

### Results and discussion

Study 2 results and reanalyses of prior studies [13, 14] are reported in Table 2. In both Study 2 samples (Austria/Germany combined and UK data), the proportion of Smiths significantly increased from light-stature to medium-stature to heavy-stature sports. This pattern is consistent with the hypothesis of Bäumler [3] and the findings from the prior two studies [13, 14]. However, our reanalysis of [13] showed that this trend effect fell short of significance (exact *p* = .066) in this first, smaller, of the two studies [13, 14], being nominally significant (*p* = .029) only in the second, larger one [14]. Across all four datasets, estimated trend parameters for the effect were of similar size (in the above order, β = 0.31, 0.19, 0.29, and 0.32), with nearly completely overlapping confidence intervals.

Conversely, both Study 2 datasets did not show decreasing proportions of Tailors from light-stature to medium-stature to heavy-stature sports, a finding inconsistent with the hypothesis of Bäumler [3] and the findings from the two prior studies [13, 14]. Reanalysis of [13] showed that the effect, largely driven by very low prevalences of Tailors in heavy-stature and medium-stature sports, was much stronger (β = -1.42, *p* = .000007) than any other effect emerging in the four samples compared here.

## General discussion

In two studies, we tested the genetic-social hypothesis of differential assortment of somatotypes on Tailor vs. Smith surnames [3, 13] with contemporary data and amended methodologies. The results of these studies are mixed, thus providing only partial support for the hypothesis.

Study 1 showed higher physical-aptitude self-ratings for dexterity-related activities (prototypical professions, sports, and hobbies combined) among Tailors, as well as corresponding higher ratings for strength-related activities among Smiths. However, while these effect were hypothesis consonant, both were small and only the latter significant. In contrast to Bäumler [3], we observed no Tailor-Smith differences in basic physique variables. Study 2 replicated evidence from two prior studies [13, 14] for an increasing prevalence of Smiths in track-and-field disciplines favoring light vs. medium vs. heavy stature in two independent samples of national elite athletes. However, we did not replicate the predicted reversed trend among athletes with the surname Tailor in the same datasets, which were large, population-based, and from two distinct language areas (Austria/Germany and UK).

Study 1 was commensurate to the sample of Bäumler [3] and thus of equal statistical power. As for Study 2, our Austrian-German sample was larger than that of [13], and our UK sample almost twice as large than that of [14], and therefore our samples had correspondingly higher analytic power. Study 1 utilized a true random sampling approach from a clearly delineated underlying population of interest, and the Study 2 samples actually were population-based; hence, validity threat due to selection bias was either low (Study 1) or could be ruled out by design (Study 2). However, with a single, non-significant exception (see Table 2), across both our studies, all obtained effects were smaller than those of analogous tests in the prior studies [3, 13, 14]. Above we have highlighted the liberties taken and post-hoc modifications made (i.e., so-called researcher degrees of freedom) with regards to operationalization, data analysis, and reporting in the course of these prior investigations. In general, “undisclosed flexibility in data collection and analysis” (see [23]) may well exaggerate any true effects. If anything, Tailor-Smith group differences likely are smaller than previously reported.

It is also interesting to note that, taking Studies 1 and 2 together, we generally seem to have replicated the predicted effects for namebearers Smiths, but not the corresponding effects for namebearers Tailors. One conceivable explanation for this distinction could be that physical occupational premises are more clearly delineated for blacksmiths (strength and gross-motor skills) than for tailors (marked strength not needed), whilst fine-motor skills certainly are necessary for both trades. Given this conjecture is viable, it would point to one inherent conceptual difficulty of the hypothesis of Bäumler [3], because of non-optimal differentiation between the two target professions, in the sense of stronger positive occupational selection for blacksmiths than for tailors.

One additional idea for interpretation of the apparently stronger association of strength and gross-motor skills with the surname Smith, as compared to any true association of dexterity and fine-motor skills with the surname Tailor, could be found in differential opportunities in assortative mating. That is, the namebearers Smith may have been more likely endogamous than the namebearers Tailor, perhaps due to their greater numbers in the population (see the different population figures in our Study 1), which, in turn, would result in larger phenotypic effects. Relatedly, it is obvious that the blacksmith occupation, due to differences in the equipment needed to work in this trade, always was more place bound than the tailor occupation. In a similar vein, such differences in the degree of sedentariness may have resulted in more pronounced patterns of endogamy and assortative mating and, in turn, in larger phenotypic effects among namebearers Smith.

An alternative, entirely different (i.e., non-genetic), and superordinate interpretation of the prior [13, 14] and our evidence could be provided by implicit egotism [24]. That is, apparently due to automatic positive self-associations, individuals tend to gravitate to others, localities, or things resembling their self. Albeit currently discussed controversially, a body of archival, field, and experimental studies suggest that, among other such effects, individuals above chance level may follow aspects of their name in some way [25, 26, 27]. One specific example of implicit egotism effects is nominative determinism, i.e., the observation that surnames seem to influence occupational choices above chance. Such effects have repeatedly been documented in research [28], by finding, for example, among librarians individuals with surname (or surname constituent) ‘Book’ or among physicians specialized in pain management individuals with surname (or surname constituent) ‘Pain’.

Applied to our topic, this could then mean that Smiths, merely because of their surname, would feel more inclined to weight-train, be preferentially drawn into heavy-physique, strength-related sports, and so forth. *Vis-à-vis* the hypothesis of Bäumler [3], the relation of this explanatory model would be one of reverse causation, i.e., in the end, Smiths would be overrepresented in strength sports because of accumulating implicit-egotism effects induced by their surname, not because of initially existing physical advantages.

Neither Bäumler’s studies [3, 13, 14] nor our studies were first and foremost designed to disentangle these alternative explanatory possibilities. Future research would, therefore, benefit from pitting these competing hypotheses against each other in appropriately devised follow-up tests. Notwithstanding that, in this context we note that the implicit-egotism hypothesis might be hard to reconcile with the differentiated findings of ours (Smith effects in both studies, but a lack of counterpart effects for Tailors in the same studies), whilst the hypothesis of stronger positive occupational selection for blacksmiths than for tailors as well as stronger positive selection for strength (and heavy-stature) sports than for endurance (and light-stature) sports would certainly be consistent with the pattern we observed. Hence, in the light of the empirical evidence accumulated so far the hypothesis of differential positive selection for occupations would appear more viable than the hypothesis of implicit egotism effects.

We envisage a number of additional, not mutually exclusive, ideas for fruitfully advancing this line of inquiry. These include: tests of further candidate surnames [14]; general population tests in rural environments (instead of metropolitan areas so far; cf. Study 1); tests of further sport types (instead of track-and-field events so far), including contemporary blacksmith/farrier contests; utilization of family-study and case-control approaches (thereby controlling for milieu similarity and familial resemblance, see [29]); and historic analyses of target surname prevalences across occupations as well as relative to the general population (although the blacksmith profession now is near-extinct, such tests appear feasible using 19th-century or early 20th-century trade directories).

Finally, bearing in mind that all general population tests so far were survey-based self-reports, it would be interesting to assess fine-motor vs. gross-motor skills and physique variables directly, although implementing the sampling frames (solely based on participants’ surnames) needed for such lab studies might be challenging. In any event, the present studies suggest that further work is necessary before firm conclusions can be drawn about Bäumler’s genetic-social hypothesis [3] of differential assortment of bodily frames among certain surnames.

## Supporting Information

S1 Dataset Study 1 dataset. (SAV)

S1 Text Study 1 Telephone Interview Text and Verbal Informed Consent Form. (DOCX)

S2 Text Study 2 National Sports Ranking Online Databases. (DOCX)
