# Supplementary material for: What’s in a Surname? Physique, Aptitude, and Sports Type Comparisons between Tailors and Smiths
Source: PLoS One. 2015 Jul 10;10(7):e0131795. doi: 10.1371/journal.pone.0131795 (PMC4498760; doi:10.1371/journal.pone.0131795)
Supplement: S1 Text — (DOCX) [file pone.0131795.s002.docx]

**English translation of standardized text (from the German original) for Study 1 telephone interviews (including verbal consent over the telephone):**

“Hello, my name is … I am a master’s thesis candidate student at the University of Vienna [departmental information]. Do I speak to Mr. …?

Mr. …: I am currently conducting telephone interviews for research, the topic of which is concerned with perceived physical aptitude for several activities, regarding professions, hobbies, and sports. We are interested in the ways in which these map onto age, height, and weight.

Your phone number has been randomly selected from the telephone directory entries. May I kindly invite you to participate in this survey, if you like, are interested, and have some time. Your participation is completely voluntarily. You can, of course, withdraw at any point during this interview, if you decide so. This will be without any consequences; specifically, I will not call you again later on.

All information you provide is strictly confidential and will only be analyzed for research purposes. All analyses will be based on groups of individuals; that is, we are interested in general trends and patterns, not in specific individual data. All data will be archived anonymously, that is, will neither be linked to your name nor to your phone number.

This telephone survey will take about 8 minutes of your time. I will ask you a total of 15 questions, namely your age, height, and current weight, and 12 further questions. In this latter part, you will be requested to state your physical aptitude prerequisites for certain activities located in the domains of professions, hobbies, or sports.

There are no foreseeable risks or discomforts with participating in this survey. Also, there are no personal benefits with participating in this survey. If you are interested in this study’s findings, I will provide you contact information to obtain these, once the study is completed.

Do you have any questions at this point? Do you agree to voluntarily participate in this telephone survey?“

[Upon agreement, i.e., verbal consent over the phone via Yes answers to these two questions, specific instructions were provided, alongside the study items, as follows:]

“May I kindly request you to assess your physical aptitude for several activities. That is, how well you think you are able to perform these activities, based on your physical aptitude prerequisites. I would like you to focus only on your physical aptitude, not on your interest in or preference for these activities. OK?

Please assess your physical aptitude according to the school-grade system, from 1 to 5. That is, 1 would indicate *very good* aptitude, whereas 5 would indicate *insufficient* aptitude. OK? Please respond honestly and spontaneously.

I now present to you at a glance 4 activities each from 3 domains (or areas of life). Then, I will repeat each single activity and would request you to assess your physical aptitude for each activity.

The first domain is about 4 professions: watchmaker, butcher, precision engineer, and brick-layer. I now read out each profession again and will then request you to assess your physical aptitude for each of these professions.“

[Questions posed and answers repeated and recorded.]

“The second domain is about 4 sports: archery, weightlifting, billiards, and rowing. I now read out each profession again and will then request you to assess your physical aptitude for each of these professions.“

[Questions posed and answers repeated and recorded.]

“The third domain is about 4 hobbies: model-making, gardening, painting, and house alteration. I now read out each profession again and will then request you to assess your physical aptitude for each of these professions.“

[Questions posed and answers repeated and recorded.]

“Finally, I would like to request you to state your age, your height, and your weight. Again, I emphasize that all the informations you provide will be handled anonymously and confidentially.“

[The data provided for age, height, and weight repeated and recorded.]

“This completes the survey. Many thanks for your time and your participation, Mr. … . Do you have any remaining questions at this point? In case you are interested in further study background and the study findings itself, or should any further questions arise, you can reach me under my e-mail address … or, alternatively, under my cell phone number … [the cell phone number from which the caller called]. Thanks again, and good bye!“
