# Supplementary material for: What’s in a Surname? Physique, Aptitude, and Sports Type Comparisons between Tailors and Smiths
Source: PLoS One. 2015 Jul 10;10(7):e0131795. doi: 10.1371/journal.pone.0131795 (PMC4498760; doi:10.1371/journal.pone.0131795)
Supplement: S2 Text — (DOCX) [file pone.0131795.s003.docx]

**Supplemental information: national sports ranking online databases (URLs)**

Germany (state athletics associations):

Baden: <http://www.blv-online.de/index.php?id=664>

Bayern: <http://www.blv-sport.de/index.php?id=91>

Berlin: <http://www.leichtathletik-berlin.de/wettkaempfe/bestenlisten/bestenlisten.htm>

Brandenburg: <http://www.nixlauf.de/>

Bremen: <http://www.leichtathletik-in-bremen.de/index.php?siteid=10>

Hamburg: <http://www.hhlv.de/wettkampf/bestenlisten>

Hessen: <http://www.hlv.de/SERVICE/bestenlisten.asp>

Mecklenburg-Vorpommern: <http://www.lvmv.de/content.php?nav=Statistik&action=Bestenlisten>

Niedersachsen: <http://www.nlv-la.de/index.php?siteid=176>

Nordrhein: <http://www.lvnordrhein.de/index.php?option=com_content&view=category&id=45&Itemid=82>

Pfalz: <http://www.lv-pfalz.de/bestenlisten.html>

Rheinhessen: <http://www.lvrheinhessen.de/?Wettkampf:Bestenliste>

Rheinland: <http://www.lvrheinland.de/93.0.html>

Saarland: <http://www.slb-saarland.com/zahlen-daten-fakten/bestenlisten/jahr/2011.html>

Sachsen-Anhalt: <http://www.lvsa.de/webKreator/index.asp>

Schleswig-Holstein: <http://www.shlv.de/index.php?option=com_wrapper&view=wrapper&Itemid=107>

Thüringen: [http://www.tlv-sport.de/wettkampfwesen/bestenlisten 50](http://www.tlv-sport.de/wettkampfwesen/bestenlisten%2050)

Westfalen: <http://www.flvw.de/leichtathletik/bestenliste.html>

Württemberg: <http://www.wlvbest.de/WLV-Bestenliste/Archiv_2011.html>

Austria (federal athletics association):

<http://www.oelv.at/lists/>

United Kingdom (UK athletics rankings):

<http://www.thepowerof10.info/rankings/>
